# Biogenic Sulfidation of U(VI) and Ferrihydrite Mediated by Sulfate-Reducing Bacteria at Elevated pH

**DOI:** 10.1021/acsearthspacechem.1c00126

**Published:** 2021-10-21

**Authors:** Luke T. Townsend, Gina Kuippers, Jonathan R. Lloyd, Louise S. Natrajan, Christopher Boothman, J. Frederick W. Mosselmans, Samuel Shaw, Katherine Morris

**Affiliations:** † Research Centre for Radwaste Disposal and Williamson Research Centre for Molecular Environmental Science, Department of Earth and Environmental Sciences, School of Natural Sciences, The University of Manchester, Manchester M13 9PL, U.K.; ‡ Centre for Radiochemistry Research, Department of Chemistry, School of Natural Sciences, The University of Manchester, Manchester M13 9PL, U.K.; § Diamond Light Source Ltd., Diamond House, Harwell Science and Innovation Campus, Didcot, Oxfordshire OX11 0DE, U.K.

**Keywords:** sulfidation, sulfate-reducing bacteria, uranium, radioactive waste disposal, GDF, EXAFS, XAS

## Abstract

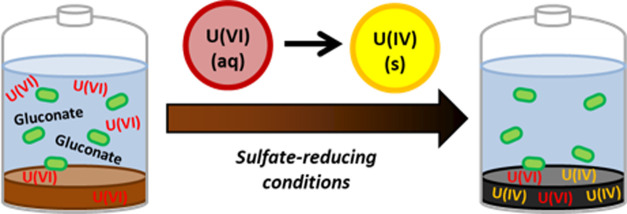

Globally, the need for radioactive waste disposal and contaminated land management is clear. Here, gaining an improved understanding of how biogeochemical processes, such as Fe(III) and sulfate reduction, may control the environmental mobility of radionuclides is important. Uranium (U), typically the most abundant radionuclide by mass in radioactive wastes and contaminated land scenarios, may have its environmental mobility impacted by biogeochemical processes within the subsurface. This study investigated the fate of U(VI) in an alkaline (pH ∼9.6) sulfate-reducing enrichment culture obtained from a high-pH environment. To explore the mobility of U(VI) under alkaline conditions where iron minerals are ubiquitous, a range of conditions were tested, including high (30 mM) and low (1 mM) carbonate concentrations and the presence and absence of Fe(III). At high carbonate concentrations, the pH was buffered to approximately pH 9.6, which delayed the onset of sulfate reduction and meant that the reduction of U(VI)_(aq)_ to poorly soluble U(IV)_(s)_ was slowed. Low carbonate conditions allowed microbial sulfate reduction to proceed and caused the pH to fall to ∼7.5. This drop in pH was likely due to the presence of volatile fatty acids from the microbial respiration of gluconate. Here, aqueous sulfide accumulated and U was removed from solution as a mixture of U(IV) and U(VI) phosphate species. In addition, sulfate-reducing bacteria, such as *Desulfosporosinus* species, were enriched during development of sulfate-reducing conditions. Results highlight the impact of carbonate concentrations on U speciation and solubility in alkaline conditions, informing intermediate-level radioactive waste disposal and radioactively contaminated land management.

## Introduction

Uranium (U) is a radionuclide of global importance due to its use within the nuclear industry, its presence as a significant component of many radioactive wastes, and its occurrence at many radioactively contaminated land sites. Currently, the globally favored management pathway for higher activity radioactive wastes containing U and other radionuclides is via an engineered geological disposal facility (GDF), which is intended to prevent the release of harmful quantities of radionuclides to the surface environment over geological time scales.^1^ As a result, U will be present in radioactive wastes emplaced within the deep subsurface, with its environmental fate significantly controlled by its speciation. Uranium speciation may be altered by microbial processes that can influence redox behavior^2−5^ and thereby induce changes in chemical form, such as dissolved or colloidal U.^6^ Additionally, many proposed intermediate-level waste (ILW) GDF systems involve the use of cement as a significant proportion of both the wasteform and, in some cases, the backfill. Here, iron (oxyhydr)oxide minerals may be present from both engineered and natural sources, including the corrosion of steel canisters and rock. Furthermore, in many ILW disposal designs, an alkaline chemically disturbed zone (CDZ) is expected to form in the near-field of a GDF due to the reaction of high-pH groundwater, which has passed through cement, with the surrounding host rock.^2,7^ The CDZ is expected to partition many radionuclides (including U) to the solid phase, via precipitation and adsorption to mineral surfaces, thereby immobilizing potential contaminants.^2^ Furthermore, a range of carbonate concentrations (∼0.2–12 mM from both natural and engineered sources)^8−10^ are expected in the latter stage of evolution of a cementitious GDF environment from sources such as biodegradation and, in some cases, in groundwaters. These differing carbonate concentrations may play a role in controlling the environmental fate of U.^11,12^ Given the effects microbial processes may have on U mobility, it is important to understand how microbial activity may impact U environmental fate in conditions relevant to this complex and evolving engineered environment.

Under oxic conditions at ambient pH, U dominates as the U(VI) uranyl moiety (UO_2_^2+^), which is usually present as soluble aqueous species, such as U(VI) carbonates.^13,14^ Consequently, U(VI) is considered as potentially more environmentally mobile compared to reduced U(IV) species, which are often present under anoxic conditions as poorly soluble uraninite (U(IV)O_2_) and/or noncrystalline U(IV).^13,15−17^ In environments where iron minerals are present, iron (oxyhydr)oxide phases have been observed to be a critical control on the environmental mobility of U(VI) by partitioning U to the solid phase either via adsorption to the surface or incorporation within the structure.^18−22^ Furthermore, U(VI) may be partitioned to the solid phase by mineralization with anions such as phosphate (PO_4_^3–^), which form poorly soluble U(VI) (and U(IV)) species.^23,24^ Phosphate may be present at significant levels in host rock environments (>3000 ppm in alkali basalts),^25^ in wastestreams (∼9.5 ppm in TBP-containing wastes),^26−28^ and in steel canisters (450 ppm).^29^

Within many radioactive wastes, U will be present at significant concentrations from a variety of wastestreams including depleted, natural, and low enriched uranium (DNLEU).^30^ As reducing conditions are expected to develop post closure of a GDF due to the exhaustion of any available oxygen,^31^ the U(VI) present in these wastes means there is potential for its reduction, and therefore alteration in environmental mobility, by abiotic or biotic processes. Under these reducing conditions, biotic processes may be driven by microbial communities depending on the availability of a range of electron donors and terminal electron acceptors.^32^ Electron donors that are generally associated with GDF systems include hydrogen,^33^ isosaccharinic acid,^34,35^ and gluconate, with this study focusing on exploring how gluconate would behave in a microbially active ILW relevant scenario. Potential electron donors and microbial growth substrates in intermediate level wastes include cement additives from the significant volumes of cementitious material due for disposal, as well as organic wastes including cellulosic materials.^36^ An illustrative electron donor for microbial growth in a potential ILW disposal environment is gluconate, a model compound for cement additives.^37^ Gluconate (C_6_H_12_O_7_) also has the ability to complex a range of radionuclides including U in both U(IV) and U(VI) oxidation states, with complexes tending to form more readily at acidic (pH 2–4)^38^ or alkaline (pH > 12) pH values.^37,39,40^ Potential electron acceptors in the deep subsurface include Fe(III) present in a variety of mineral phases and sulfate ions (SO_4_^2−^) that may be abundant in deep groundwaters (for example, ∼0.3–3 mM sulfate in Sellafield groundwaters).^41−43^ The presence of electron donors in the waste and sulfate as a potential electron acceptor can stimulate sulfate-reducing bacteria (SRB) that, in turn, produce sulfide (H_2_S/HS^–^).^42,44−47^ In the presence of bioavailable Fe(III)-bearing minerals, Fe(II) may also be microbially produced via Fe(III)-reducing bacteria or some SRB.^48,49^ However, microbial reduction rates are, slowed under alkaline conditions, in particular the SO_4_^2−^/HS^–^ redox couple, as the energy yield for this couple decreases when approaching pH ∼10 or higher.^32,34^

U(VI) mobility can be impacted by microorganisms via a variety of different processes, including biosorption to the cell surface (coordinated by ligands such as phosphates and organic acid moieties^50−52^), biomineralization (including precipitation as U phosphate minerals^14^), and enzymatically mediated reduction of U(VI) to U(IV) with the formation of poorly soluble noncrystalline U(IV) and/or nanoparticulate uraninite.^13,15,24,53−56^ A range of Fe(III)- and sulfate-reducing bacteria are capable of U(VI) reduction via enzymatic electron transfer.^4,5,48^ Here, the periplasmic enzyme, cytochrome *c*_3_, is pivotal in reducing U(VI) to U(IV) in SRB.^3,5,57,58^ The exact pathway is unknown but a single electron transfer from U(VI) to form an unstable intermediate U(V), which then may undergo disproportionation to U(VI) and U(IV), is most likely.^59−61^

In terms of abiotic reactions, the presence of reducing agents may impact the fate of U, as U(VI) is known to undergo abiotic reduction by HS^–^ in solution and by Fe(II) at mineral surfaces, consequently reducing its environmental mobility.^62−67^ In addition, in systems containing Fe(III)-mineral-containing, reaction with sulfide is known to produce Fe(II) which transforms the Fe(III)-(oxyhydr)oxides to Fe(II)-bearing phases, such as mackinawite (FeS).^68,69^ U(VI) reduction by reaction with sulfide generally takes place in solution and forms solid uraninite-like phases.^62,70,71^ Fe(II)-mediated U(VI) reduction to U(IV) (generally as U(IV)O_2_) can also take place either via electron-transfer mineral surfaces^65−67^ or by direct interaction with Fe(II)-bearing mineral phases present, such as mackinawite and magnetite (Fe_3_O_4_),^63,72,73^ and it is notable that U(VI) reduction is slowed with elevated levels of carbonate.^62,70,74^ Recent abiotic laboratory sulfidation studies have highlighted that transient U(VI) remobilization can occur during sulfidation of U(VI)/iron (oxyhydr)oxide-containing systems.^71,75−77^ Remobilization of U(VI) under sulfidation conditions has also been observed in field studies, where Fe(III)- and sulfate-reducing conditions have been induced to remediate soluble U(VI).^47,78^ Following microbially mediated U(VI) reduction, Anderson et al. observed an unexpected release of U(VI) into solution during the change from Fe(III)-reducing to sulfate-reducing conditions.^78^ Such findings suggest that the biogeochemical fate of U is complex under sulfidic conditions and the sulfidation process itself may lead to significant, if transient, changes in speciation and possible implications for its mobility and fate.

In many deep geological disposal scenarios, reducing conditions are expected to develop as resaturation occurs post GDF closure due to both the exclusion of air and the onset of metal corrosion in the waste environment. Additionally, electron donors may be present as intermediate level wastes contain organic materials, including cellulose, decontamination agents, and/or waste stabilizers. These electron donors may stimulate the host microbial community to develop a range of anaerobic metabolic processes, including Fe(III) and sulfate reduction, that may impact the fate of contaminants, including U.^3,13,46,79^ As a result, the potential range of biogeochemical processes operating in alkaline conditions needs to be understood to further underpin predictions of the environmental fate of U. Here, biogenic sulfidation experiments were performed under elevated pH conditions (pH ∼9.5) to improve understanding of the fate of U(VI) in systems that reflect the microbial processes that may occur in scenarios relevant to ILW disposal. Experiments included low and high carbonate concentrations of 1 and 30 mM, respectively. In addition, the impact of Fe(III) on U fate in these systems was explored. Gluconate, a model compound for cement additives in a cementitious ILW GDF, was used as a carbon source. These experiments used an anaerobic sulfate-reducing microbial consortium enriched from an alkaline analogue field site (Harpur Hill, U.K.) under elevated pH (pH ∼9.5) conditions. The microbial consortium was used to probe the potential for gluconate-mediated biotic sulfate reduction under alkaline conditions and to explore its fate on uranium speciation.^35^ The results highlight both the impact of carbonate at high concentrations in maintaining U(VI) solubility and the microbially mediated changes to the system that drive U immobilization as both U(VI) and U(IV) phosphate species under low carbonate, sulfate-reducing conditions.

## Experimental Methods

### Sediment Characteristics

Sediment samples and surface waters were collected from a legacy lime working site in Buxton, U.K.^32,80^ Sediment samples were taken from a depth of ∼20 cm, with the pH values of the sediment-associated water and surface water being 9.4 and 11.5, respectively. The sediment was selected because of its high pH geomicrobiology and has been used as a model system with relevance to cementitious ILW disposal scenarios.^32,34,35,80^ Both the sediment and water were kept in the dark, under anaerobic conditions as appropriate, and at 4 °C until used.

### Ferrihydrite Preparation

Ferrihydrite was synthesized following the method of Cornell and Schwertmann.^81^ Briefly, Fe(III) chloride was dissolved in deionized water (DIW) before neutralizing with NaOH to pH 7. The resulting red-brown precipitate was washed with DIW five times. The product was stored under anaerobic conditions for a maximum of 1 month prior to use. Characterization was carried out using X-ray diffraction (XRD), and the total iron concentration was determined using a modified ferrozine assay.^82,83^ Ferrihydrite was used as it is an environmentally relevant, reactive, bioavailable source of Fe(III).

### Enrichment of Sulfate-Reducing Bacteria

Sulfate-reducing enrichment cultures for experimental incubations were obtained using a 1% (v/v) sediment inoculum added to modified Postgate medium B that omitted sodium lactate, yeast extract, and thioglycolate (Section S1).^84,85^ In addition, 6 mM Na-gluconate was added to the medium as the sole electron donor and carbon source. Enrichment cultures were incubated at 20 °C in the dark. During robust sulfate reduction (indicated by the formation of a dark black precipitate), a 1% (v/v) inoculum was transferred to fresh medium, until after seven consecutive transfers, a stable enrichment culture for experimentation was obtained.

### Biogenic Sulfidation Experiment with U(VI)

Autoclaved and degassed modified Postgate B medium (40 mL) was inoculated with 1% (v/v) of the sulfate-reducing microbial enrichment in 50 mL of serum bottles. The modified Postgate medium B contained elevated sulfate (∼12 and 15 mM in the high and low carbonate systems, respectively) and phosphate (∼4 mM) (see Section S1). Each experiment contained Na-gluconate (6 mM) as the sole electron donor and carbon source, NaHCO_3_ at either low or high concentrations (1 or 30 mM, respectively), U(VI)O_2_^2+^ (0.1 mM), and ferrihydrite ([Fe(III)_total_] = 1 mmol/L slurry) for the experiments containing Fe(III). Experiments were run in triplicate with the following additions: (i) U(VI)-only, (ii) U(VI) + Fe(III), and (iii) Fe(III)-only. Experiments were run for between 5 and 6 weeks (35 days for the high carbonate system, 42 days for the low carbonate system). Controls containing no added electron donor or autoclaved sterile cultures were prepared alongside (see Section S1).

### Geochemical Analysis

Samples were taken periodically for pH, Eh, U(VI)_(aq)_, Fe_(aq)_, HS_(aq)_^–^, and solid-phase analysis using anaerobic, aseptic techniques. For aqueous analyses, slurry samples were centrifuged at 16 160*g* for 10 min, the aqueous phase was separated and preserved for analysis through the addition of fixing reagents (acidification to 2% HNO_3_ for U(VI)_(aq)_ and Fe_(aq)_; zinc sulfide precipitation for HS_(aq)_^–^),^86^ and solid samples were frozen at −80 °C. Aqueous analysis was performed by inductively coupled plasma mass spectrometry (ICP-MS) on a Perkin-Elmer Optima 5300 DV, for U and Fe, and by using a methylene blue assay for aqueous HS^–^ (using the calibration standard Radiello RAD171).^86^ Sulfate, thiosulfate, and organic acids were analyzed by ion-exchange high-performance liquid chromatography (IE-HPLC) using a Dionex ICS5000 Dual Channel on Chromatograph, fitted with a Dionex AS-AP autosampler and a CD20 conductivity detector.

### Solid-Phase Analysis

X-ray absorption spectroscopy (XAS) was used to determine the U speciation at selected time points. Samples were produced by collecting biomass- and mineral-containing precipitates by centrifugation at 16 160*g* for 5 min. The resulting solids were then diluted in cellulose under anaerobic conditions to a final U concentration of up to ∼1 wt %. A pressed pellet was then formed, which was mounted, frozen at −80 °C, and stored under these conditions prior to analysis. Samples were then transported under liquid N_2_ conditions in a dry shipper to Diamond Light Source for analysis on the B18 beamline. XAS spectra were obtained in a liquid nitrogen cryostat from the U L_III_ edge (17166 eV) in fluorescence or transmission mode using a 36-element Ge detector. Data was collected to a *k*-range of ∼14, and fitting was typically to a *k*-range of 12. All sample edge positions were calibrated using the data obtained from an in-line Y reference foil. Data reduction and fitting of the EXAFS spectra were performed using Athena and Artemis with FEFF6.^87^

Samples were prepared for environmental scanning electron microscopy (ESEM) by washing the slurry with deionized deoxygenated water, before depositing it on an aluminum pin stub, and drying anaerobically. The instrument used was an FEI XL30 ESEM-field emission gun (ESEM-FEG) operating at 15 kV in high vacuum mode (10^–5^–10^–6^ mbar) with an EDAX Gemini energy-dispersive X-ray spectroscopy (EDS) system.

### 16S rRNA Gene Sequencing

16S rRNA gene sequencing was performed^88^ with the Illumina MiSeq platform (Illumina, San Diego, CA) using a Roche “Fast Start High Fidelity PCR System” (Roche Diagnostics Ltd., Burgess Hill, U.K.). The used primers were the forward 515F (5′-GTG YCA GCM GCC GCG GTA A-3′) and reverse 806R (5′-GGA CTA CHV GGG TWT CTA AT-3′), targeting the V4 hypervariable regions for 2 × 150-bp paired-end sequencing. For full details on analysis and bioinformatics, see Kuippers et al.^88^

## Results and Discussion

### Biogenic Sulfidation Experiment with U(VI)

**Figure 1:**
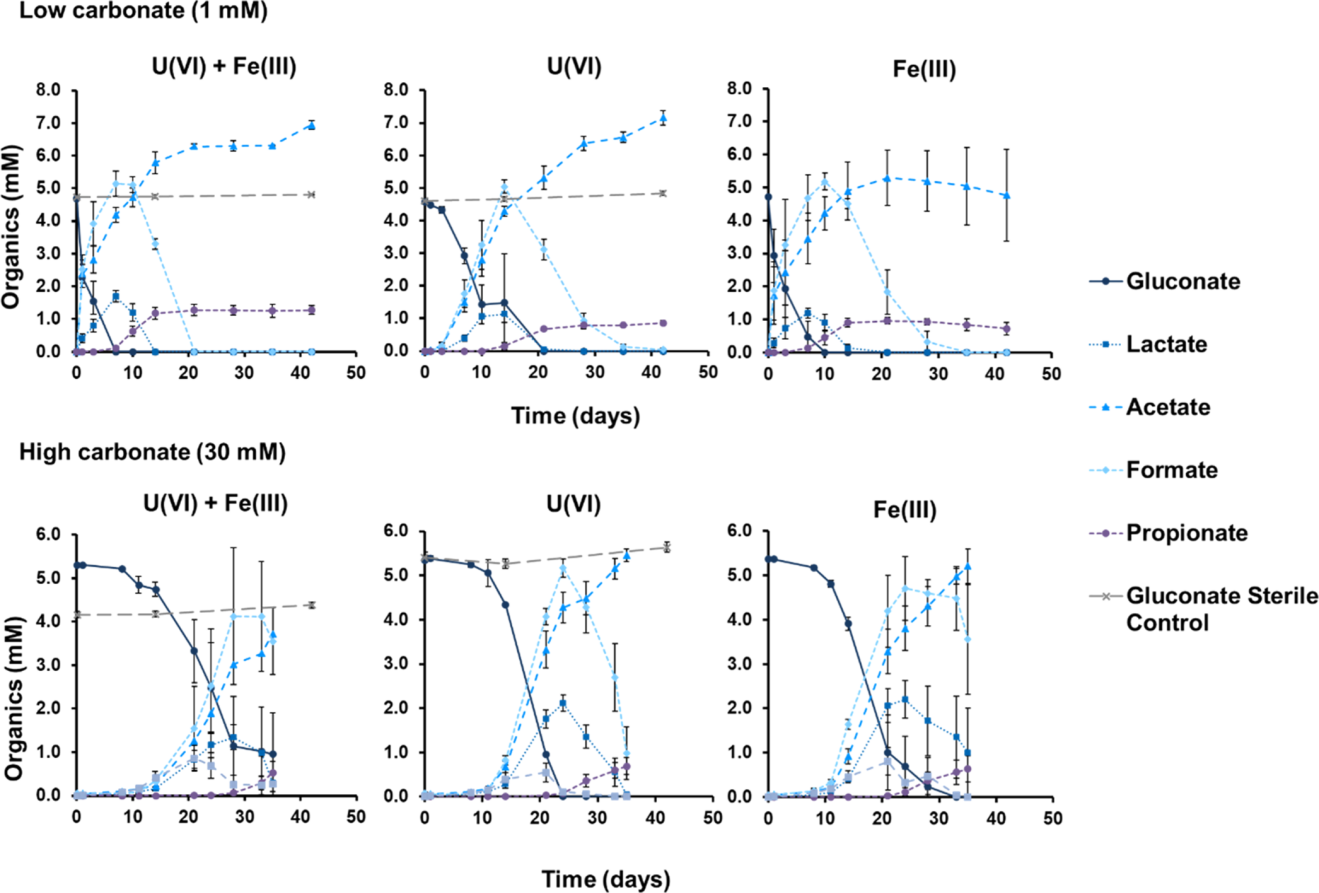
Ion chromatography data for the organics present in the microbially active cultures under low and high carbonate conditions with corresponding sterile control gluconate concentrations.

For the biogenic sulfidation experiment, enrichment cultures were set up under sulfate-reducing conditions using an enrichment from an alkaline legacy lime working sediment as the inoculum. In the microbially active cultures at low and high carbonates (U(VI)-only, U(VI) + Fe(III), and Fe(III)-only), gluconate was removed from solution (Figure 1). Gluconate concentrations remained constant in sterile controls (Figure S2-1), indicating that gluconate was removed only in the microbially active experiments. The degradation products from gluconate metabolism included volatile fatty acids (VFAs), predominantly formate and acetate, and lower amounts of lactate, propionate, and pyruvate (Figure 1). Acetate and propionate accumulated in the cultures until the end of the experiment, while other VFAs were further metabolized. All active microbial cultures darkened throughout the duration of the experiment, consistent with the development of reducing conditions. The U(VI)-only cultures changed from white to gray, with cultures amended with U(VI) + Fe(III) or Fe(III) changing from ferruginous to black indicating the development of Fe(III) and/or sulfate reduction (Figure S1-1).

**Figure 2:**
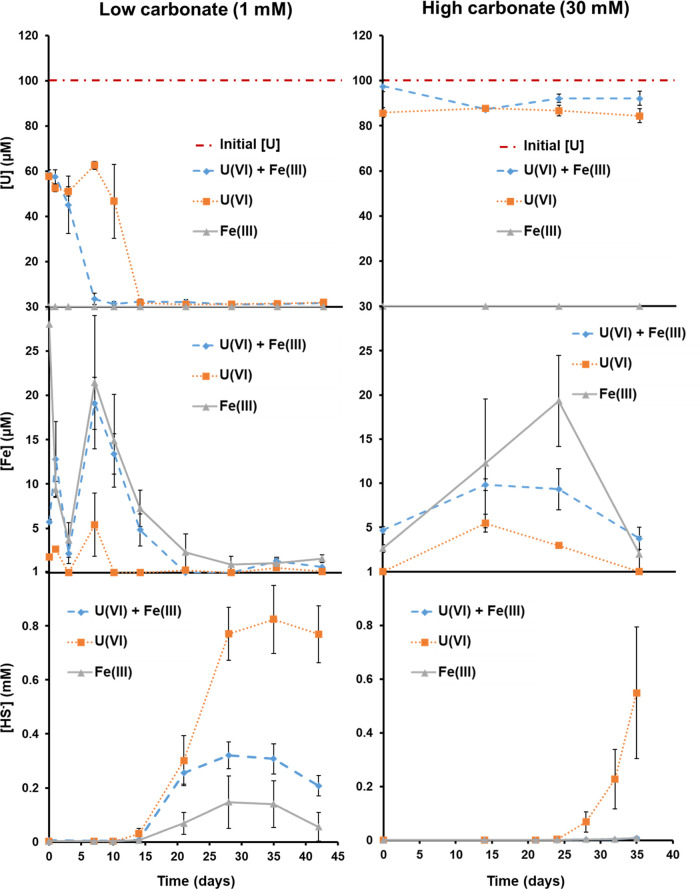
Aqueous geochemical data from the low and high carbonate enrichment culture experiments. Total U and Fe concentrations were measured using ICP-MS. Aqueous sulfide concentration was measured using the methylene blue assay.

Sulfate reduction was indicated by the removal of ∼1 mM SO_4_^2–^ from solution in the active microcosms (from initial concentrations of ∼12 and ∼15 mM in the high and low carbonate systems, respectively) and ingress of HS_(aq)_^–^ (Figures 2, S2-2, and S2-3). Given that the experiment had excess electron donor, this suggests that time may be limiting the system in terms of sulfate reduction. Interestingly, sulfate reduction proceeded at a faster rate under low carbonate conditions (after day 10) compared with that under the high carbonate conditions (after day 21). This is likely due to the high carbonate conditions inhibiting sulfate reduction through buffering of the pH to ∼9.6 (Figure S2-4), close to the reported upper pH limit of microbial sulfate reduction.^32^ Sterile and no electron donor controls showed no removal of sulfate from solution over the duration of the experiment (Figure S2-1).

In terms of redox potential, the low carbonate systems became reducing at a faster rate (∼−120 mV at day 14), reaching strongly negative Eh values (−250 to −330 mV) by day 21 (Figure S2-4). These values are broadly in line with the redox couple for sulfate reduction at high pH.^89^ The low carbonate systems exhibited a decrease in pH, from 9.6 to ∼7.5, between days 7 and 14, before stabilizing around pH 8 for the remaining duration of the experiment. The acidification of the microbially active cultures is presumably due to accumulation of VFAs from microbial degradation of gluconate and/or acidification from CO_2_.^34^ High carbonate systems became reducing (∼−128 mV) at 28 days, with a final Eh at 35 days of −200 to −320 mV, again broadly consistent with sulfidic conditions (Figure S2-4). This suggests a delay in the development of sulfate reduction due to the elevated pH compared to the low carbonate system.^32^ In contrast to the microbially active systems, the abiotic controls maintained pH values between 9.4 and 9.8 throughout the experiment, with a slightly downward trend in pH with time, presumably due to equilibration processes (Figure S2-5).

Under high carbonate conditions, almost no U(VI) was removed from solution in the U(VI)-containing cultures with the concentration around 89.2 ± 5.3 μM (∼88% total U) throughout the experiment, despite the clear evidence for development of sulfidic conditions at the end point (day 35; Figure 2). Similar results were observed in the high carbonate sterile controls where no sulfate reduction was observed (86.6 ± 4.6 μM; ∼85% total U; Figure S2-6). The retention of U(VI) in solution was likely due to the dominance of U(VI) species, presumably U(VI)-triscarbonate, which is known to be recalcitrant to reduction.^74,90,91^ Uranium solution speciation was investigated via fluorescence spectroscopy on the sample end point supernatants (Figures S3-1 and S3-2), with spectra confirming close matches with the published U(VI)-triscarbonato species.^90,92^ Interestingly, despite the presence of significant reducing potential in the form of aqueous Fe (presumably Fe(II)), solid Fe(II), and sulfide ([Fe_(aq)_]_max_ = ∼18 μM at day 14, U(VI) + Fe(III); [HS_(aq)_^–^]_max_ = ∼0.57 mM at day 35, U(VI)-only) (Figure 2), no significant U(VI) removal or reduction was observed. This suggests that the stable aqueous uranyl carbonate complexes formed at high pH were recalcitrant to reduction by enzymatic and abiotic means which is consistent with the past work.^70,74,93^ Additionally, the high pH may also be impeding the rate of development of bioreduction for U(VI) and sulfate as previously discussed.^32^

In the low carbonate cultures, the aqueous U concentration at the start of the experiment (*t* = 0 days) was 58.0 ± 2.0 μM (∼57% total U), with comparable values seen in the low carbonate sterile experiments (41.1 ± 8.0 μM; ∼40% total U; Figure S2-6). Interestingly, the low carbonate, no electron donor (no gluconate) control, which had biomass present, showed a further drop in aqueous U(VI) concentrations with time (26.4 ± 2.4 μM; ∼25% total U; Figure S2-6), indicating that in the microbially active experiments, gluconate may have been complexing and solubilizing the U(VI) in the systems.^37,38^ The cultures were modeled at both high and low carbonate concentrations in PHREEQC (using the SIT database)^94^ to further explore their predicted U solubility (Section S2-2). Here, modeling of key aqueous inorganic species was performed at pH values 7.5 and 9.5 to explore U(VI) solubility. For the low carbonate system, the thermodynamic modeling results suggested that the majority of U(VI) was likely to remain soluble, with modest saturation of clarkeite (sodium uranate) at pH 9.5 and some oversaturation of crystalline U(VI) phosphates and clarkeite at pH 7.5 predicted. Clarkeite presence in these systems would be considered unlikely as it is expected to be a high-temperature phase.^95^ Despite this, more recent work has shown that high pH, GDF-relevant conditions can induce clarkeite-like phase formation.^6^ The combined modeling and geochemical data suggested that the immediate removal of ∼50% U(VI) from solution in the active and sterile low carbonate cultures may be due to modest oversaturation of U(VI) and/or sorption to biomass.^13^

Over time, the low carbonate microbially active cultures showed removal of the remaining aqueous U from solution by day 14 in the U(VI)-only and by day 7 in U(VI) + Fe(III) cultures (Figure 2). U(VI) removal from solution in U(VI) + Fe(III) cultures coincided with the increase in aqueous Fe concentrations, presumably as soluble Fe(II) (∼18 μM) from biogenic Fe(III)-reduction at day 7. The observed removal of U from solution presumably reflects either enzymatic or abiotic reduction of U(VI) to U(IV), with abiotic removal likely associated with U(VI) reacting with Fe(II) to form U(IV) at mineral surfaces (Figure 2). In the low carbonate U(VI)-only cultures, significant U removal was not observed until the ingress of aqueous sulfide from approximately day 10. Again, the observed removal may be due to enzymatic reduction of U(VI) or abiotic reductive precipitation of U(VI) to U(IV) by HS_(aq)_^–^.^4,62,70,96^

### Solid-Phase Analysis of Low Carbonate Cultures

To further investigate the speciation of U in the solid phase of the low carbonate system where U had been removed from solution, a combination of XAS and ESEM imaging was performed on selected samples. ESEM was used to image the end point samples of low carbonate U(VI)-amended experiments (both with and without added Fe) (Section S6), and XAS samples were taken at days 3 and 42 from the same experiments.

**Figure 3:**
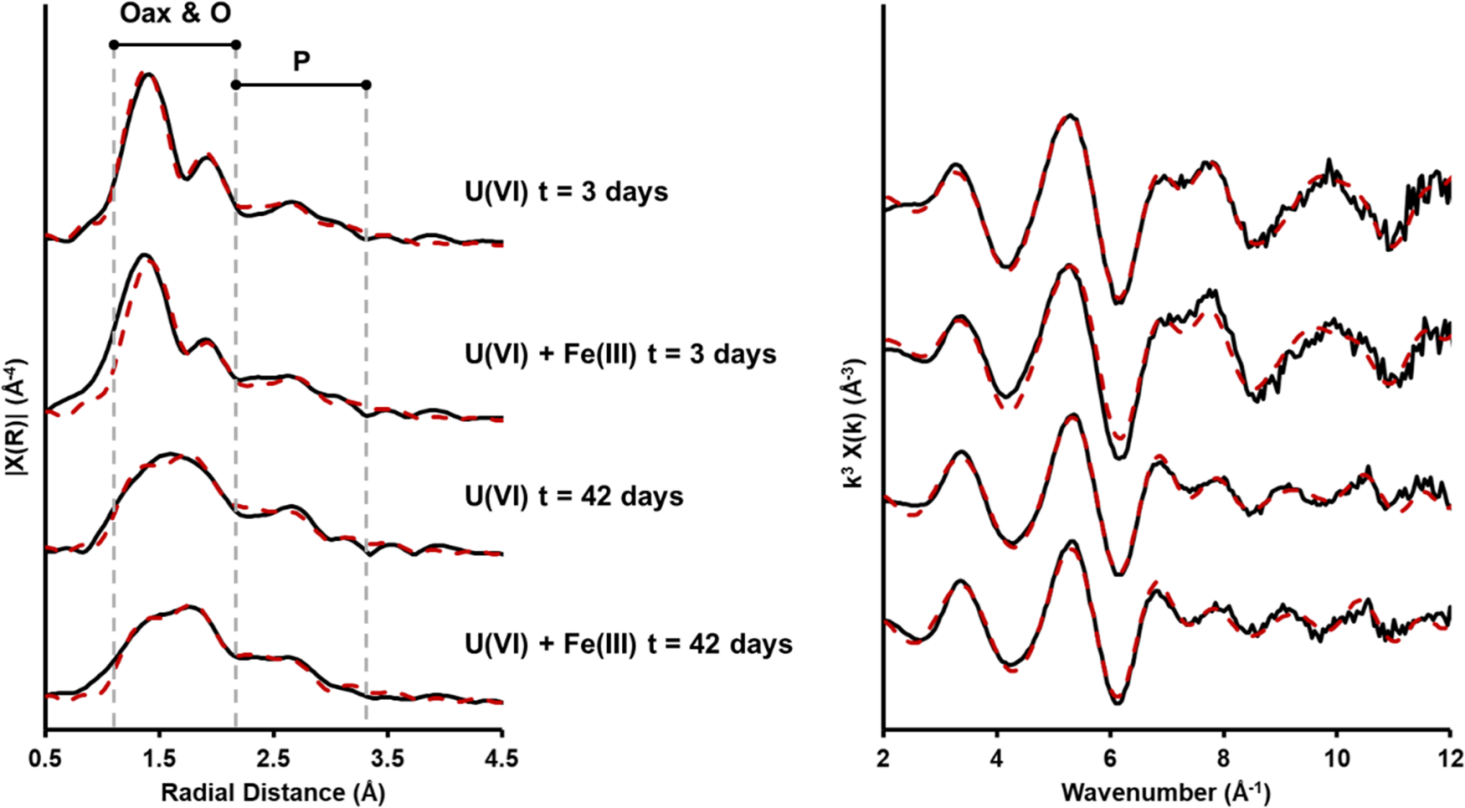
Fourier transform (left) of the *k*^*3*^-weighted EXAFS (right) U L_III_ edge EXAFS for the microbially active low carbonate solid-phase samples with and without Fe(III).

Analysis of the U L_III_ edge XANES spectra edge positions of the low carbonate system, both with and without added Fe(III), showed a general trend of reduction from U(VI) to U(IV) from day 3 to the end point (Figure S5-1). Comparison of the edge positions with U(VI) and U(IV) standards suggested a mixed U(IV)/U(VI) system for the day 42 samples in both U(VI) and U(VI) + Fe(III) cultures (Figure S5-1). Interestingly, when compared to the U-only system, the presence of Fe(III) (as ferrihydrite) did not seem to impact the speciation of U throughout the experiment (through adsorption to the mineral surface),^19,97^ with similar U XANES and EXAFS spectra obtained for the with and without added Fe(III) experiments. The best model for the EXAFS spectra at day 3, for both U(VI)-only and U(VI) + Fe(III) experiments, included ∼1.8 oxygen (O) backscatterers at ∼1.80(1) Å, ∼3 O backscatterers at 2.32(2) Å, ∼3.6 O backscatterers at 2.48(2) Å, ∼1.4 phosphorus (P) backscatterers at 3.13(2) Å, and 1.2 P backscatterers at 3.63(4) Å (Figure 3 and Table 1). This model is consistent with predominantly a U(VI) uranyl species coordinated by phosphate ions in a mixture of monodentate (P shell at 3.62(1) Å) and bidentate (P shell at 3.12(1) Å) coordination environments, suggesting initial sorption of a fraction of the U(VI) to biomass as a U(VI) phosphate species,^98,99^ or precipitation of a solid U(VI) phosphate precipitate. Due to similar bond distances and coordination numbers across a variety of different U phosphate phases (Table S5-1),^17,23,50,98−102^ it was not possible to further assign a specific structure in this system. Despite this, the geochemical data, PHREEQC modeling, and EXAFS fitting models confirm that in both U(VI)-only and U(VI) + Fe(III) experiments, U(VI) is either immediately adsorbed to the biomass or precipitated from solution as a U(VI) phosphate species.

**Table 1:** Fitting Parameters for the EXAFS Data for the Microbially Active Low Carbonate Solid-Phase Samples with and without Fe(III)^a^

			path						
time point (days)	experiment	parameter	O_ax_	O1	O2	P1	P2	*E*_0_	*R*-factor
3	U(VI)	CN	1.8	3	3.5	1.5	1.2	8.3(17)	0.011
		σ^2^ (10^–3^)	2(1)	2(2)	3(2)	4(2)	3(3)		
		*R* (Å)	1.80(1)	2.32(2)	2.48(2)	3.13(2)	3.63(4)		
	U(VI) + Fe(III)	CN	1.7	2.8	3.7	1.3	1.2	10.0(17)	0.009
		σ^2^ (10^–3^)	2(1)	3(2)	4(2)	3(2)	3(3)		
		*R* (Å)	1.81(1)	2.32(2)	2.48(2)	3.13(2)	3.63(3)		
42	U(VI)	CN	0.7	4.2	3.5	1.5	1.2	3.0(21)	0.013
		σ^2^ (10^–3^)	2(2)	3(2)	2(1)	3(2)	3(4)		
		*R* (Å)	1.76(2)	2.28(2)	2.44(2)	3.08(2)	3.58(4)		
	U(VI) + Fe(III)	CN	0.7	4.5	3.3	1.5	1.3	4.7(19)	0.011
		σ^2^ (10^–3^)	3(2)	4(1)	3(2)	3(2)	4(4)		
		*R* (Å)	1.78(2)	2.31(2)	2.47(2)	3.10(2)	3.61(4)		

a The amplitude reduction factor (S_0_^2^) was set as 1 for all fits. CN denotes the coordination number (fixed during fitting), *R* denotes the interatomic distances, σ^2^ denotes the Debye–Waller factor, and *E*_0_ denotes the shift in energy from the calculated Fermi level.

The samples at day 42, for both U(VI)-only and U(VI) + Fe(III), produced EXAFS models indicating the presence of both U(VI) and U(IV) species. The best fit model for both U(VI)-only and U(VI) + Fe(III) enrichment cultures included 0.7 O backscatterers at 1.77(1) Å, ∼4.3 O backscatterers at ∼2.30(2) Å, ∼3.4 O backscatterers at ∼2.46(2) Å, 1.5 P backscatterers at ∼3.09(2) Å, and ∼1.3 P backscatterers at ∼3.60(4) Å (Figure 3 and Table 1). These models are consistent with a mixture of U(VI) and U(IV) species, with phosphate ions coordinated in both monodentate and bidentate orientations. The reduction in the coordination number from the day 3 to the day 42 sample of the O_ax_ component at ∼1.8 Å (from 1.8 to ∼0.7) indicates that the reduction of ∼50–60% U(VI) to U(IV) had occurred. This is consistent with aqueous U(VI) being reductively precipitated as U(IV) as bioreduction progressed potentially against a baseline of U(VI) phosphate precipitation/sorption in the early experiment (Figure 2). Overall, throughout the experiment, the best model fits produced for the EXAFS spectra included phosphorus-based ligands, likely present in the experimental medium/biomass (Figure 3).

ESEM imaging was used to further investigate the U precipitate from the U(VI)-only and U(VI) + Fe(III) experiments. In the U(VI) + Fe(III) end point sample (Figure S6-1), three different morphologies were identified, indicated by the EDS spot analysis numbers 1–3. The backscattered image (Figure 6-1A) highlighted an area (spot 1) that was shown to be enriched with U, compared to spots 2 and 3. The morphology of this spot matched well with the hydroxyapatite-like phase seen in the U(VI)-only end point sample (Figure S6-2). Spot 3 highlighted a similar morphology to the hydroxyapatite-like phase but showed additional enrichment of Fe and S, suggesting the formation of amorphous FeS (mackinawite) phases that did not strongly associate with U. EDS mapping showed U to be strongly enriched in a phase containing mainly P and O (spot 1), when compared to the other present phases (such as FeS) (Figure S6-1B). This suggests that following the onset of reducing conditions in the system, U(IV) preferentially coprecipitates in Ca^2+^- and PO_4_^3–^-rich areas. ESEM analysis of the U(VI)-only end point sample showed a separate U- and P-enriched phase highlighted in the backscattered image (Figure S6-2A, spot 1).

Considering both the EXAFS fitting models and the ESEM and EDS analyses, the U(IV) component in the end point samples was likely a ningyoite-like (CaU^IV^(PO_4_)_2_·2H_2_O) inorganic phase or noncrystalline U(IV) associated with phosphate (likely from biomass as seen in the previous work).^50,56,101^ Previous work has shown that both noncrystalline U(IV), including ningyoite-like phases, and nanouraninite may be present through the formation and growth of U(IV) phases under bioreducing conditions.^24,103^ However, the lack of long-range order in the EXAFS data in this study’s systems (for example, a lack of U–U interatomic distance) does eliminate the likely presence of significant amounts of uraninite and/or crystalline U-phosphates over the relatively short time frames of the experimental incubation. Additionally, noncrystalline U(IV) phosphates are also reported either via direct binding of U(IV) to cell membranes or through bioreduction and biomineralization, with the EXAFS fitting models from our experiments matching well with these past studies (Table S5-1).^17,24,50,56,101^ The similarities in bond lengths for U(VI) and U(IV) phosphate species does introduce limitations on the amount of detailed speciation information that can be obtained for U(VI, IV) phosphates. However, from XAS analysis, geochemical data, PHREEQC modeling, and consultation of the literature (Table S5-1),^17,23,24,50,98,100,101^ it can be determined that the U(VI) phosphate species are likely sorbed to biomass and the U(IV) portion of the experiment is likely present as phosphate-coordinated noncrystalline U(IV).

As previously discussed, XAS data for the low carbonate system indicate that the proportion of U(VI) reduced to U(IV) is ∼50–60%. This additional reduced U(IV) in the day 42 sample compared to that in the day 3 sample is in line with the amount of U(VI) that was present in solution at the start of the experiment (0–3 days) in the low carbonate systems. Therefore, the U(VI) present in solution at the start of the experiment appears to be amenable to reductive precipitation either enzymatically or abiotically to poorly soluble and poorly ordered U(IV) phosphate phases. In contrast, the ∼40% of U(VI) that was immediately partitioned to the solid phase in the day 3 time point as U(VI) phosphate species appears to be recalcitrant to reduction by Fe(II) and HS^–^ over the relatively short time scales investigated. This suggests that solid-phase U(VI) phosphates in the environment may be recalcitrant to reduction under the conditions of this study. Overall, this suggests that any available U(VI)_(aq)_ may be reduced by either direct enzymatic or indirect biotic processes and, in a phosphate-rich environment, is likely to form U(IV) phosphate phases in agreement with previous studies.^17,24^

Regardless of whether U(VI) is abiotically or biotically reduced, the enhanced removal of uranium under low carbonate concentrations and elevated pH experiments confirms that microbially driven processes cause reductive precipitation of U. This is in line with previous findings in similar systems that investigated the effects of both carbonate concentration and pH on microbial reduction rates of U(VI).^2,74,104−106^

### Microbial Community Analysis

**Figure 4:**
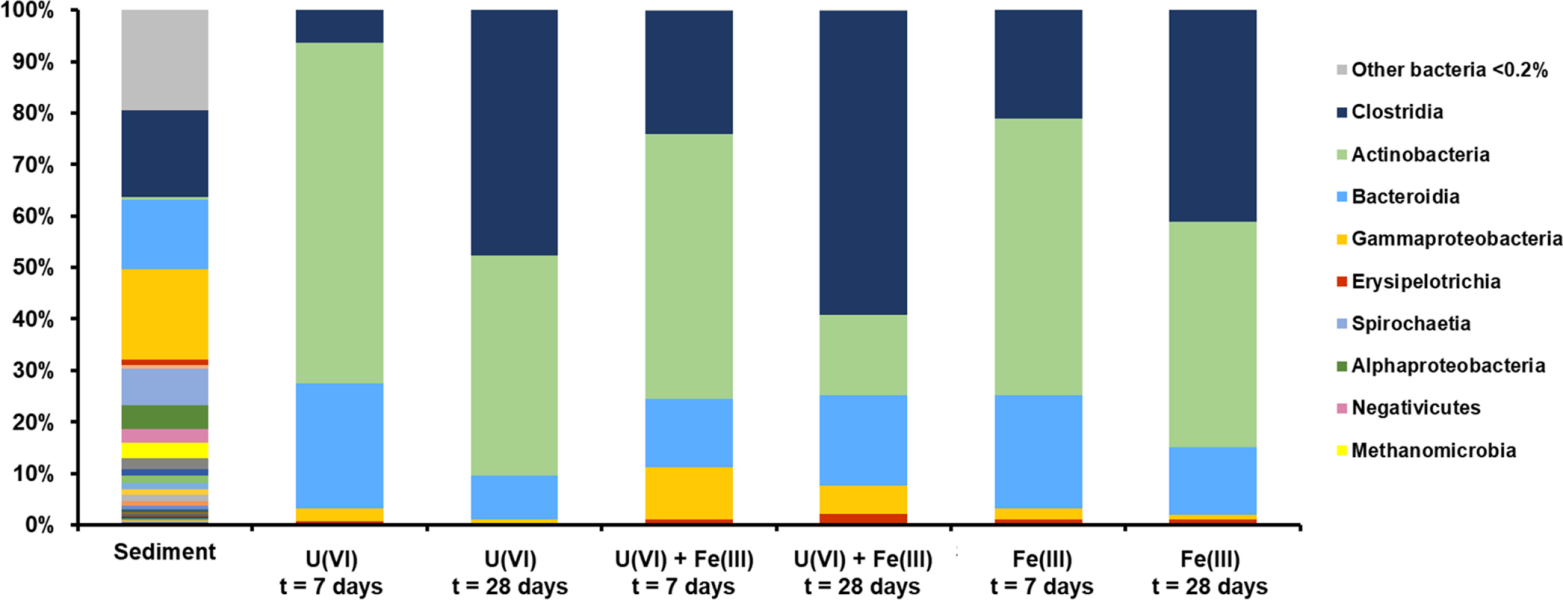
Microbial phylogenetic classes of 16S rRNA sequencing results in the low carbonate system with a cutoff at >0.2% abundance. Abundances <0.2% are summarized in group “other bacteria”.

16S rRNA gene sequencing was performed to study changes in the microbial enrichment community after incubation with U(VI) (Figures 4 and S4-1–S4-3). Compared to the complex background microbial community (>570 operational taxonomic units (OTUs)), the sulfate-reducing, gluconate-enriched consortium used for these experiments showed an order-of-magnitude decrease in species diversity (50–65 observed species) at both low and high carbonate concentrations (Figures S4-1 and S4-3). Focusing on the low carbonate system where U(VI) was removed completely, the cultures were dominated by Gram-positive bacteria comprising mainly of species from the classes Clostridia and Actinobacteria and lower percentages from the Bacteroidia, Gammaproteobacteria, and Bacilli. In the early stages of the incubation (day 7), all enrichments were dominated (29–62% of total sequences) by a bacterium most closely affiliated with *Corynebacterium faecal* (100% sequence similarity), a facultative anaerobic Gram-stain-positive bacterium known to ferment glucose but not gluconate.^107^ Another enrichment in the early stages of the incubation comprised sequences affiliated with *Parabacteroides chartae* (strain NS31-3; 100% sequence similarity), a Gram-negative bacterium that is able to use a wide range of sugars for its metabolism.^108^ Typical fermentation products of this bacterium are lactate, propionate, formate, and acetate,^108^ all of which were observed in the low carbonate experiment. As the incubations progressed, the relative percentage of Clostridia species increased throughout the treatments from day 7 (3–12% of total sequences) to day 28 (26–39% of total sequences). Overall, Clostridia were the most diverse class with 62 different OTUs identified in the enrichments. After 28 days of incubation, most sequences were most closely associated with the isolate *Desulfosporosinus fructosivorans* (type strain 63.6 F^T^; 98.8% sequence similarity), an anaerobic, spore-forming sulfate-reducing bacterium that can couple sulfate reduction to lactate oxidation.^109^ The increase in sequences of *Desulfosporosinus* species coincided with sulfide accumulation and removal of formate and lactate from solution, and was consistent with the coupling of sulfate reduction to lactate oxidation.^109^ The succession of species during the course of incubation indicates that a complex microbial community was involved in gluconate fermentation and degradation, which was coupled to sulfate reduction.

In contrast to the low carbonate system, the microbial community in the high carbonate system was dominated by Gram-negative bacteria, including members from the Gammaproteobacteria, and a small enrichment of Deltaproteobacteria (Figure S4-2). In all cultures from the high carbonate system, the most dominant organism (43% and 46% of sequences in U(VI) + Fe(III) and U(VI)-only, respectively, at day 10) belonged to an OTU most closely affiliated with a Gram-negative *Pseudomonas* species (strain KR2-15, 100% sequence similarity). Consistent with minimal sulfate reduction in the high carbonate system, sequences that were affiliated with known SRB, including sequences affiliated with *Desulfomicrobium* species, decreased with incubation time.

## Conclusions

Overall, these findings suggest that very high carbonate conditions could give rise to predominantly aqueous U(VI) carbonate species that are recalcitrant to partitioning to the solid phase via the pathways explored here, despite microbial metabolism of gluconate and ingrowth of Fe(II) and HS^–^ being observed. At lower carbonate concentrations, microbial Fe(III) and sulfate reduction strongly influence U speciation, with results suggesting that any aqueous U(VI) may be partitioned to the solid phase as poorly ordered reduced U(IV) phosphates. While this study did not explore whether reduction of U(VI) takes place via an indirect process, for example, via microbially produced Fe(II) and HS^–^, or via direct enzymatic reduction, under low carbonate conditions expected in calcium-rich subsurface environments, biogeochemical processes will have the capacity to immobilize U in the solid phase. Such information is essential in gaining a greater understanding of uranium environmental chemistry and informing the safety case associated with the disposal of radioactive waste and contaminated land management.

## Supporting Information Available

Methodology, visual observations, and consortium analysis; additional geochemical data; PHREEQC modeling; fluorescence spectroscopy; microbial community analysis; addition XAS data and analysis; and ESEM data and analysis (PDF)
