# Supplementary material for: Biogenic Sulfidation of U(VI) and Ferrihydrite Mediated by Sulfate-Reducing Bacteria at Elevated pH
Source: ACS Earth Space Chem. 2021 Oct 21;5(11):3075–86. doi: 10.1021/acsearthspacechem.1c00126 (PMC8607498; doi:10.1021/acsearthspacechem.1c00126)
Supplement: Supplementary file 1 — sp1c00126_si_001.pdf [file sp1c00126_si_001.pdf]

Supporting Information

Biogenic sulfidation of U(VI) and ferrihydrite mediated by sulfate-reducing bacteria at elevated pH

Luke T. Townsend, [ǂ] *Gina Kuippers,* [ǂ] *Jonathan R. Lloyd,* [ǂ] *Louise S. Natrajan,* [§] *Christopher Boothman,* [ǂ] *J. Frederick W. Mosselmans,* Samuel Shaw,* [ǂ] *and Katherine Morris*,* [ǂ]

[ǂ]Research Centre for Radwaste Disposal and Williamson Research Centre for Molecular Environmental Science, Department of Earth and Environmental Sciences, School of Natural Sciences, The University of Manchester, Manchester M13 9PL, UK

[§]Centre for Radiochemistry Research, Department of Chemistry, School of Natural Sciences, The University of Manchester, Manchester M13 9PL, UK

*Diamond Light Source Ltd., Diamond House, Harwell Science and Innovation Campus, Didcot, Oxfordshire, OX11 0DE, UK

Contents

Section S1: Methodology & Observations 3

Section S2: Geochemical Data 5

Section S3: Fluorescence Spectroscopy 9

Section S4: Microbial Community Analysis 11

Section S5: Additional XAS Data & Analysis 13

Section S6: ESEM Data & Analysis 15

References 17

Section S1: Methodology & Observations

All reagents used throughout this study (including mineral/media preparations and microcosm experiments) were of ACS grade or higher (purity ≥95 %).

Table S1-1 Experimental set up of all culture experiments performed in this study.

Carbonate (mM)	Experiment Label	Repeats	Enrichment (1% inoculum)	Sulfate (~13.5 mM)	Gluconate (~6 mM)	U(VI) (100 μM)	Ferrihydrite ([Fe(III)] = 1000 μM)
1	U(VI) + Ferrihydrite	3	X	X	X	X	X
	U(VI)	3	X	X	X	X	
	Ferrihydrite	3	X	X	X		X
	Sterile with Ferrihydrite	3		X	X	X	X
	Sterile	3		X	X	X	
	No electron donor	1	X	X		X	
30	U(VI) + Ferrihydrite	3	X	X	X	X	X
	U(VI)	3	X	X	X	X	
	Ferrihydrite	3	X	X	X		X
	Sterile with Ferrihydrite	3		X	X	X	X
	Sterile	3		X	X	X	

Table S1-2 The ingredients (in addition to those shown in Table S1-1) for the composition of the amended Postgate B medium used for the experiments. Compositions of Vitamin and Mineral mixes are provided in Table S1-3.

Ingredient	Concentration (L^{-1})	Molarity (mM)
$MgSO_4$	2.0 g	16.6
Na_2SO_4	0.5 g	3.5
$CaSO_4$	1.0 g	7.3
KH_2PO_4	0.5 g	3.7
Vitamin Mix	10 mL	-
Mineral Mix	10 mL	-

Table S1-3 Mineral and vitamin mix compositions.

Mineral mix composition		Vitamin mix composition	
**Ingredient**	**Concentration (g/L)**	**Ingredient**	**Concentration (g/L)**
Nitrilotriacetic acid	1.50	Biotin	2.00
$MgSO_4$	3.00	Folic acid	2.00
$MnSO_4.H_2O$	0.50	Pyrodoxine HCl	10.00
NaCl	1.00	Riboflavin	5.00
$FeSO_4.7H_2O$	0.10	Thiamine	5.00
$CaCl_2.2H_2O$	0.10	Nicotinic acid	5.00
$CoCl_2.6H_2O$	0.10	Pantothenic acid	5.00
$ZnCl_2$	0.10	Vitamin B-12	0.10
$CuSO_4.5H_2O$	0.01	*p*-aminobenzoic acid	5.00
$AlK(SO_4)_2.5H_2O$	0.01	Thiotic acid	5.00
H_3BO_3	0.01		
$NaMoO_4$	0.03		
$NiCl_2.6H_2O$	0.02		
$Na_2WO_4.2H_2O$	0.03		

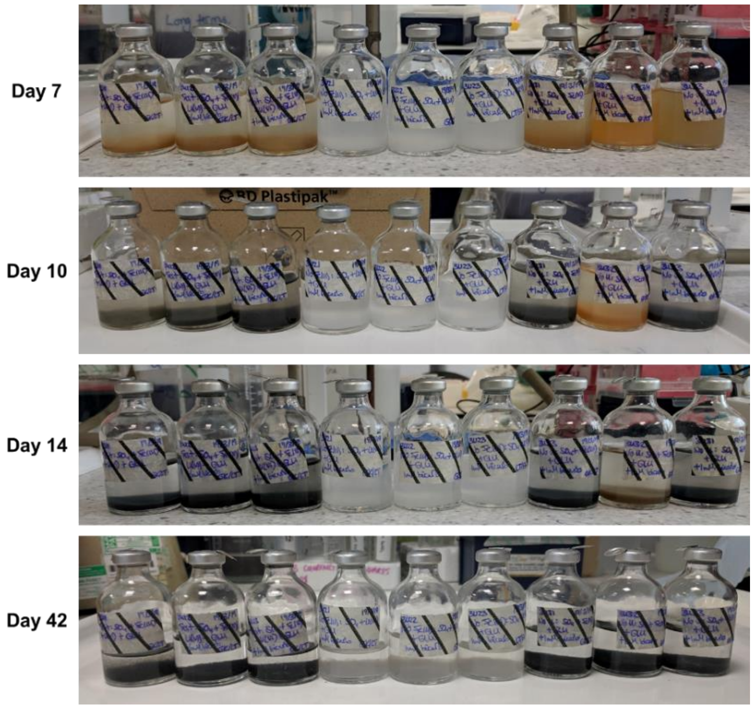

Figure S1-1 Observed colour change of the key cultures throughout the experiment under low carbonate conditions.

Section S2: Geochemical Data

Section S2-1: Additional aqueous geochemical data

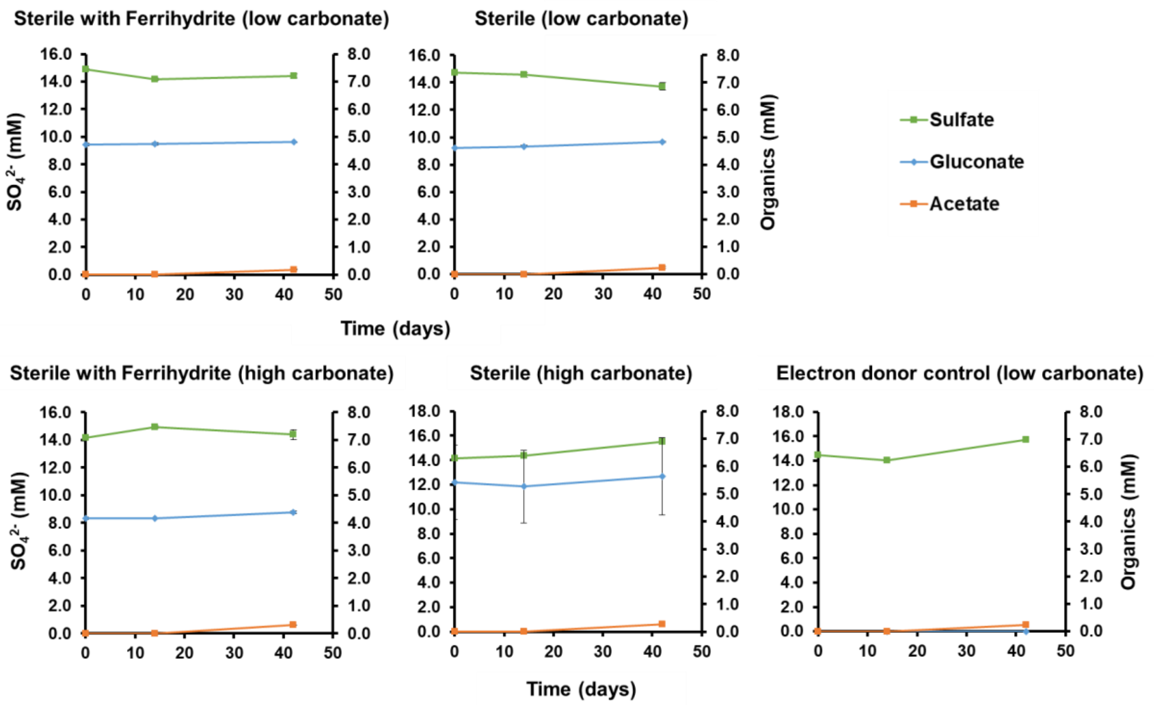

Figure S2-1 Ion chromatography data for the control experiments showing sulfate and organic concentrations. No other organics were detected in these systems than gluconate and acetate.

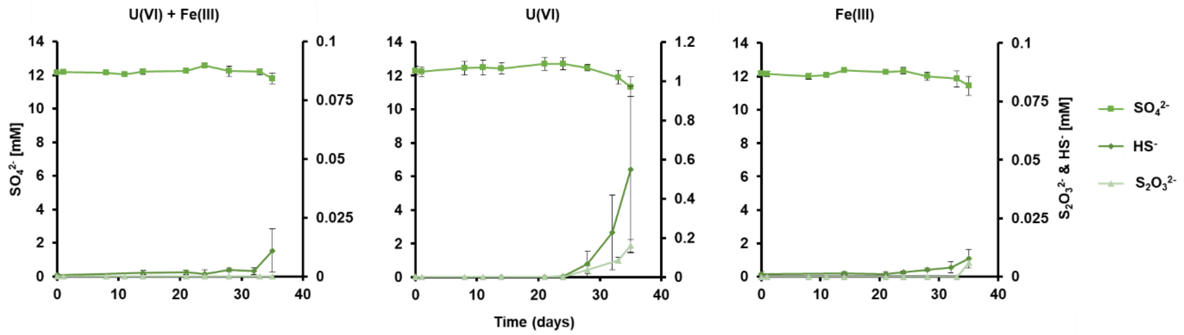

Figure S2-2 Ion chromatography data (SO_4^{2-}, $S_2O_3^{2-}$, HS^-) for the key cultures under high carbonate conditions.

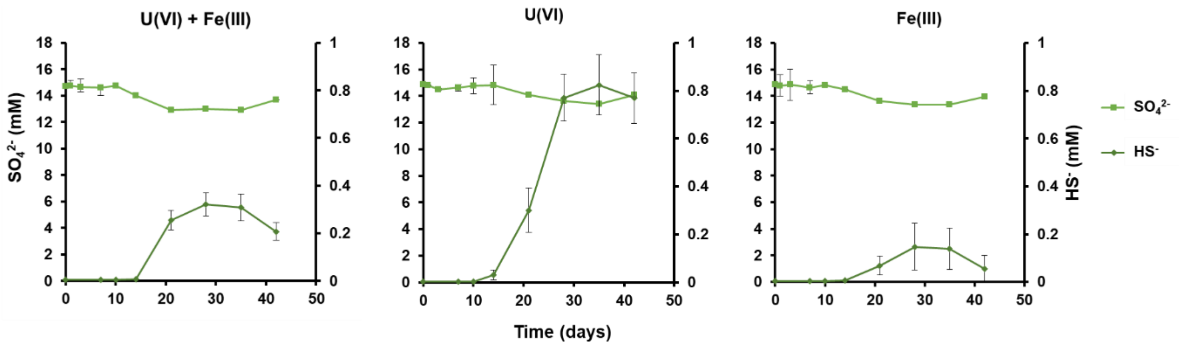

Figure S2-3 Ion chromatography data (SO_4^{2-}, HS^-) for the key cultures under low carbonate conditions. No thiosulfate was detected in these systems.

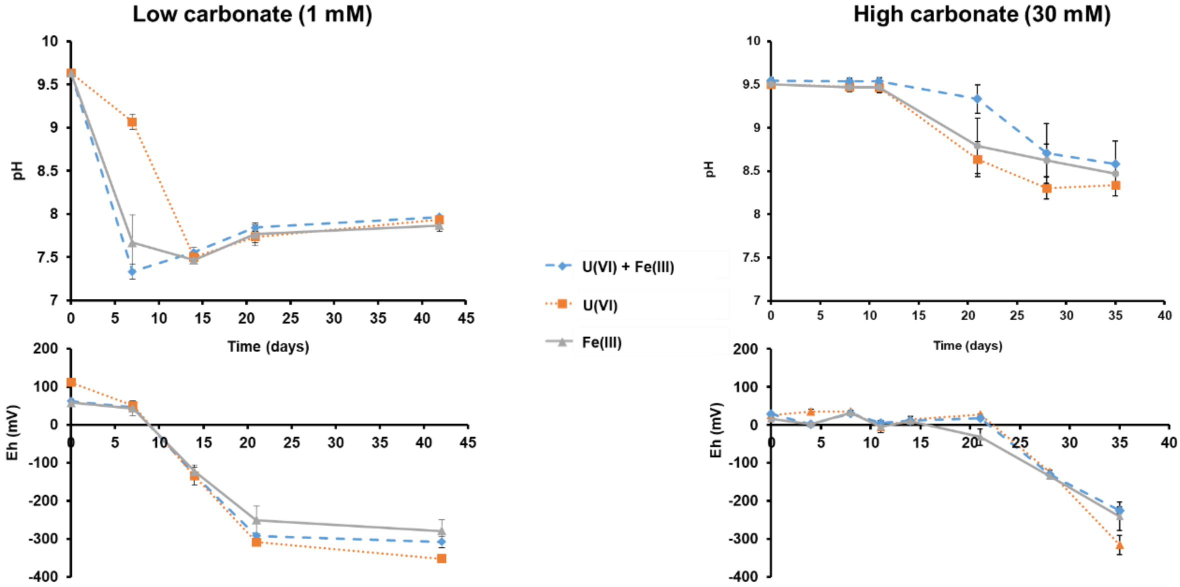

Figure S2-4 pH & Eh values for the key culture experiments under both high and low carbonate conditions.

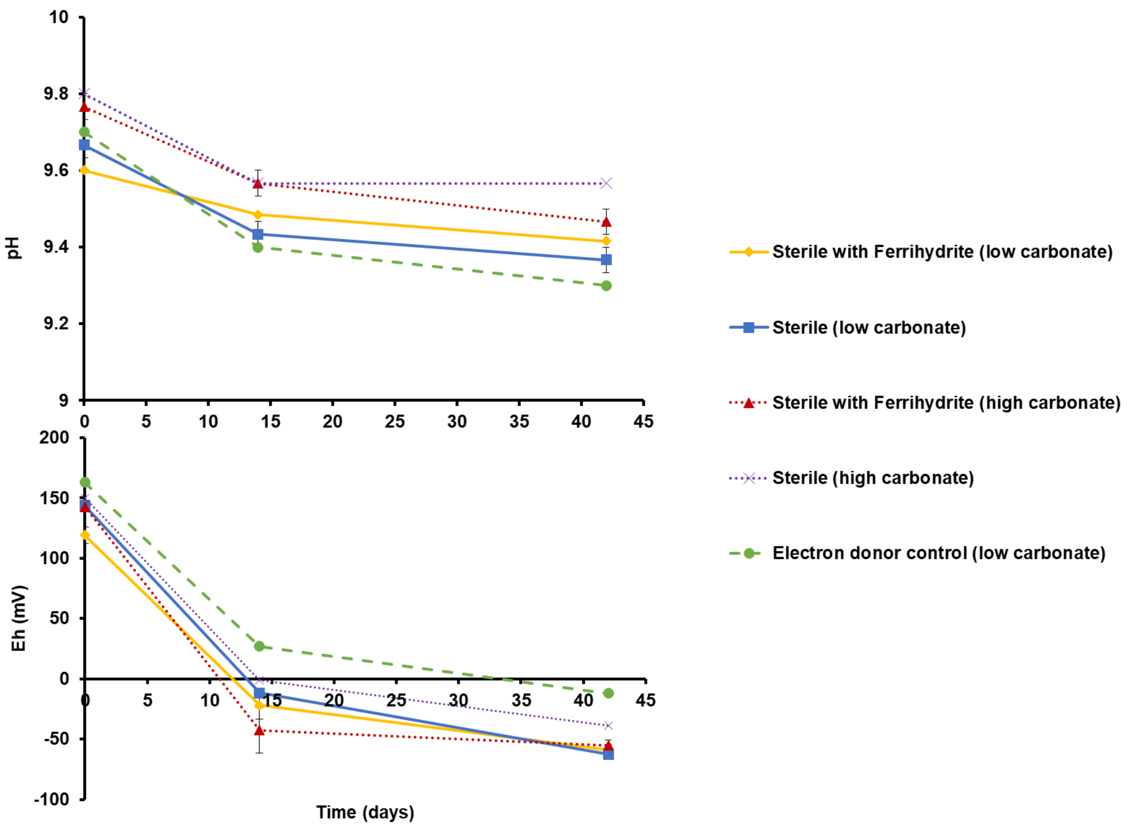

Figure S2-5 pH & Eh values for the control experiments under both high and low carbonate conditions.

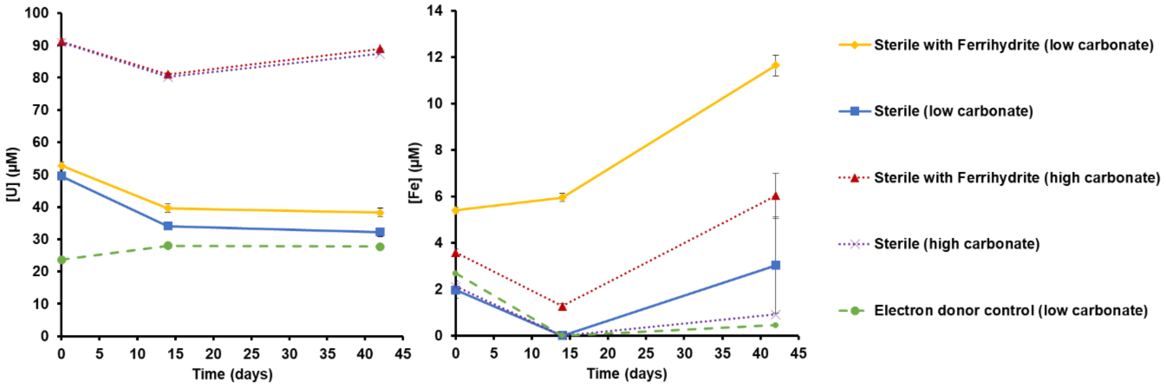

Figure S2-6 U and Fe concentrations for the control experiments under both high and low carbonate conditions.

Section 2-2: PHREEQC Modelling

An example of the simplified solution (with key inorganic species present) used to model the solubility of U(VI) in the low carbonate systems is shown below:

```
SOLUTION 1
    temp      25
    pH        7.5
    pe        0
    redox     pe
    units     mmol/kgw
    density   1
    P         3.7
    U(6)      0.055
    Glu       6
    S(6)      13.5
    C(4)      1
    Na        6 charge
    -water    1 # kg
```

Table S2-1 Composition of starting solutions for the low and high carbonate experiments according to PHREEQC. The abundance of each U(VI) species is given as a percentage (%) of the total U in solution.

U(VI) species (as listed in PHREEQC)	Low carbonate (1 mM)	High carbonate (30 mM)
UO2(CO3)3-4	99.13	99.99
UO2(CO3)2-2	0.57	0.01
UO2(OH)3-	0.19	-

Table S2-2 Key solid phases in the output file produced from the PHREEQC modelling using the example input file provided above.

Phase (as listed in PHREEQC output file)	Saturation index	
	pH 7.5	pH 9.5
(UO2)3(PO4)2:4H2O (cr)	6.65	-1.99
(UO2)3(PO4)2:6H2O (s)	2.70	-5.94
Clarkeite (Na(UO2)O(OH))	1.27	3.01

Section S3: Fluorescence Spectroscopy

Steady state emission spectra were recorded on an Edinburgh Instrument FP920 Phosphorescence Lifetime Spectrometer equipped with a 450 watt steady state xenon lamp and a red sensitive photomultiplier in peltier (air cooled) housing, (Hamamatsu R928P). Samples were loaded in sealed borosilicate NMR tubes and frozen in liquid nitrogen in a finger Dewar flask modified to fit into the spectrometer (Edinburgh Instruments) before the spectra were recorded.

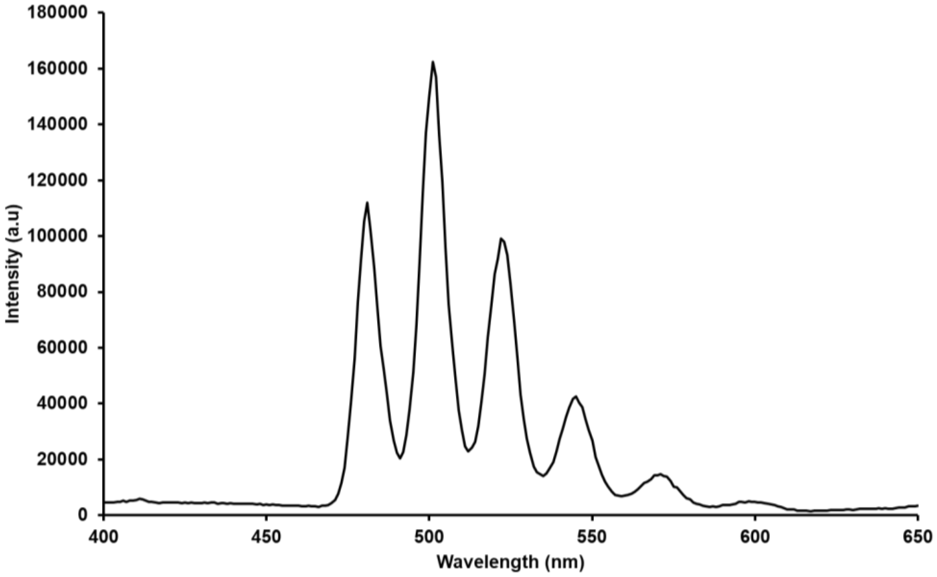

Figure S3-1 The fluorescence emission spectrum for the high carbonate solution sample for U(VI) system (t = ~35 days). Emission spectrum measurements were taken with an excitation wavelength of 280 nm, recorded at 77 K and are an average of 3 scans.

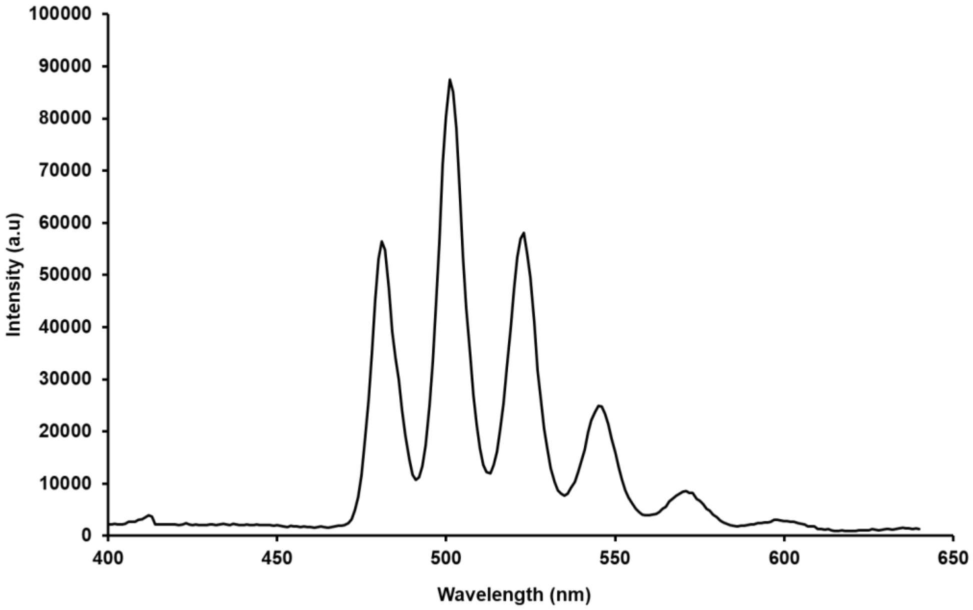

Figure S3-2 The fluorescence emission spectrum for the high carbonate solution sample for U(VI) + Fe(III) system (t = ~35 days). Emission spectrum measurements were taken with an excitation wavelength of 280 nm, recorded at 77 K and are an average of 3 scans.

Section S4: Microbial Community Analysis

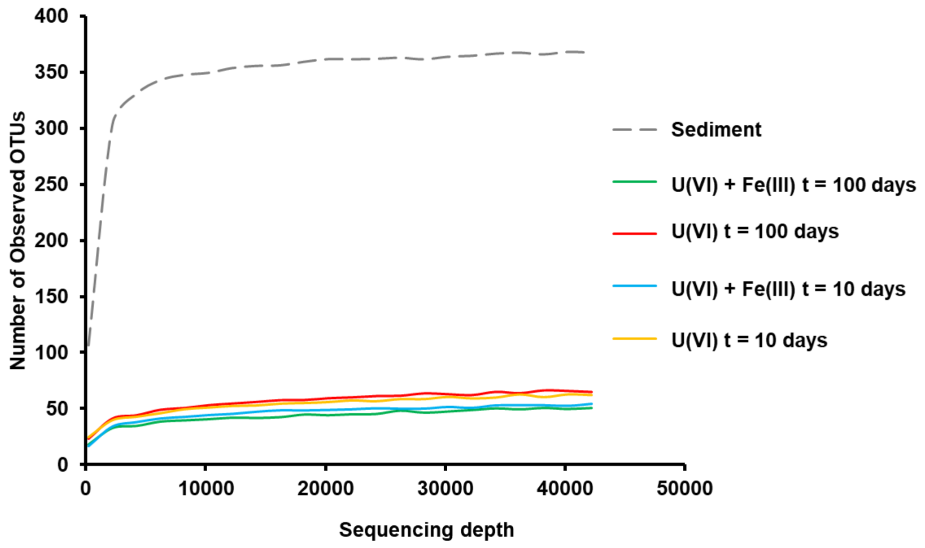

Figure S4-1 Alpha rarefaction curve showing microbial diversity in the sediment compared to the enrichments at high carbonate concentrations.

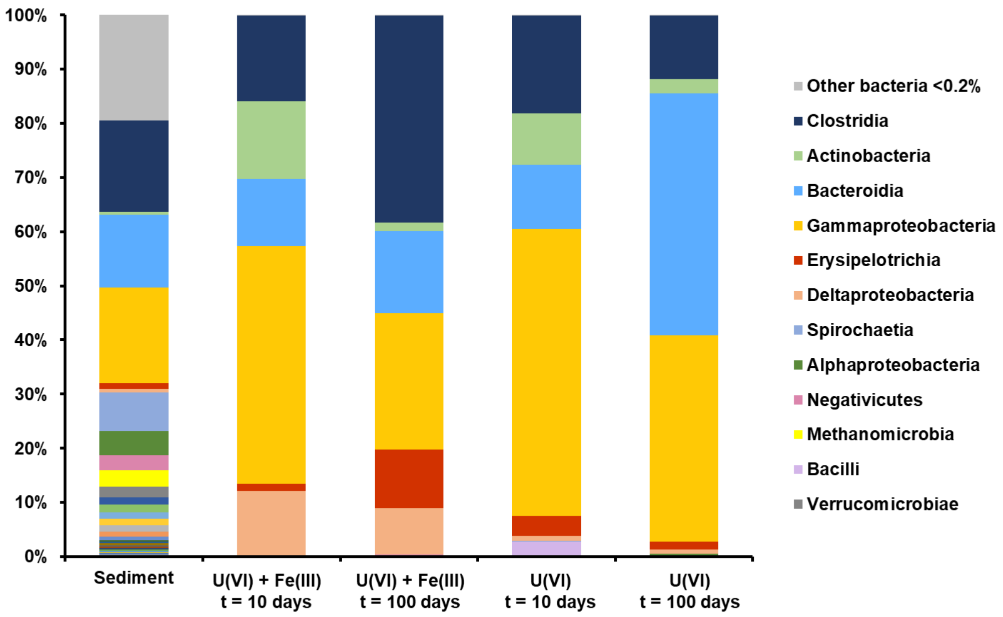

Figure S4-2 16S rRNA gene sequencing results from the high carbonate cultures of the U(VI) only and U(VI) + Fe(III) treatments, showing the microbial community by phylogenetic class in comparison to the sediment.

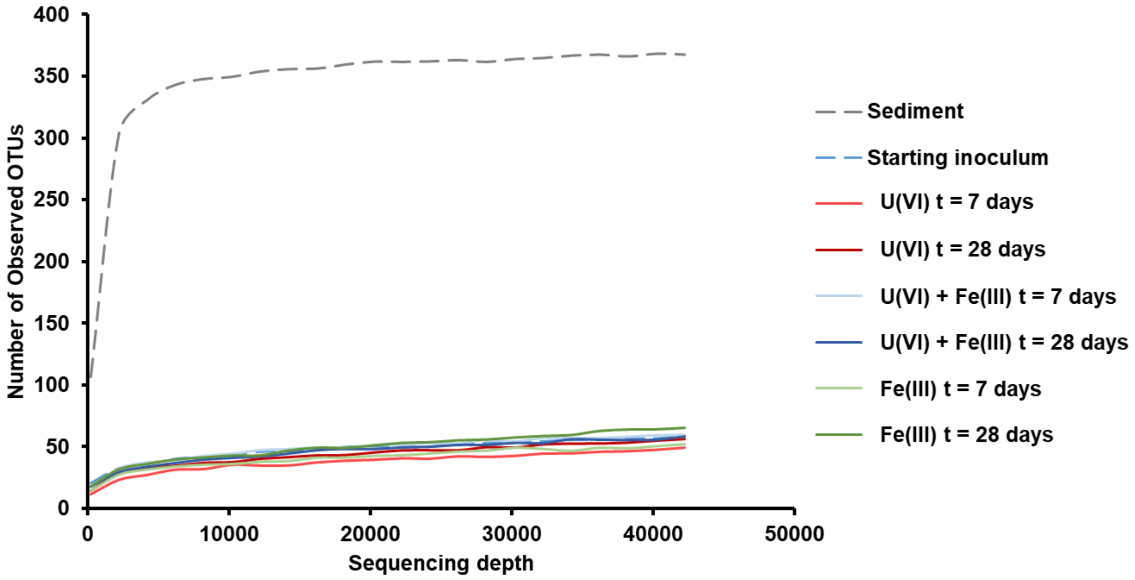

Figure S4-3 Alpha diversity rarefaction curves showing species diversity in sediment compared to enrichments at low carbonate concentration.

Section S5: Additional XAS Data & Analysis

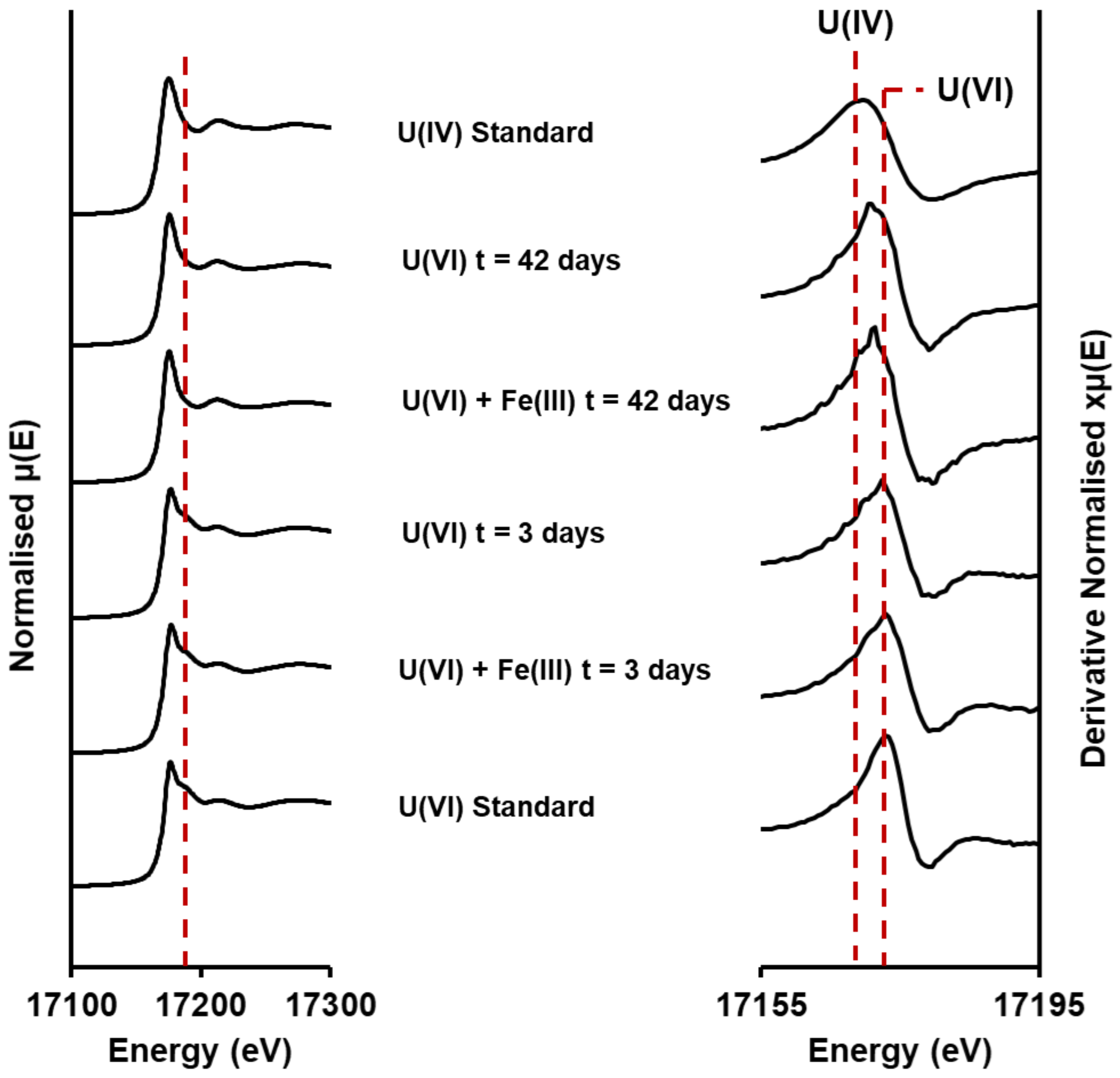

Figure S5-1 The XANES spectra for the microbially active low carbonate solid samples with and without Fe(III) at time points t = 3 and 42 days. Left shows the normalised μ(E) spectra with a line showing the characteristic 'shoulder' peak for U(VI). Right shows the first derivative of the μ(E) spectra, with U(IV) and U(VI) edge positions shown. The standards used are U(VI) adsorbed to ferrihydrite and nanoparticulate U(IV)O_2.

Table S5-1 Comparison of the average bond lengths of U(IV) and U(VI) phosphate species from the literature and this study, using the U(VI) + Fe(III) cultures results as an example.

**U oxidation state**	**Study**	**Path bond length (Å)**				
		O_{ax}	O1	O2	P1	P2
U(VI)	This study (U(VI) + Fe(III) t = 3 days)	1.77	2.31	2.48	3.13	3.63
U(IV)/U(VI) mix	This study (U(VI) + Fe(III) t = 42 days)	1.77	2.29	2.44	3.09	3.59
U(IV)	Alessi et al. (2014)	-	2.28	2.44	3.14	3.69
	Boyanov et al. (2011)	-	2.29	2.47	3.16	3.72
	Bargar et al. (2013)	-	2.37	-	3.13	3.64
	Newsome et al. (2015)	-	2.27	2.42	3.12	3.64
U(VI)	Beazley et al. (2009)	1.78	2.27	-	-	3.60
	Singh et al. (2012)	1.78	2.34	2.48	-	3.60
	Mehta et al. (2016)	1.80	2.31	2.48	3.11	3.60

Section S6: ESEM Data & Analysis

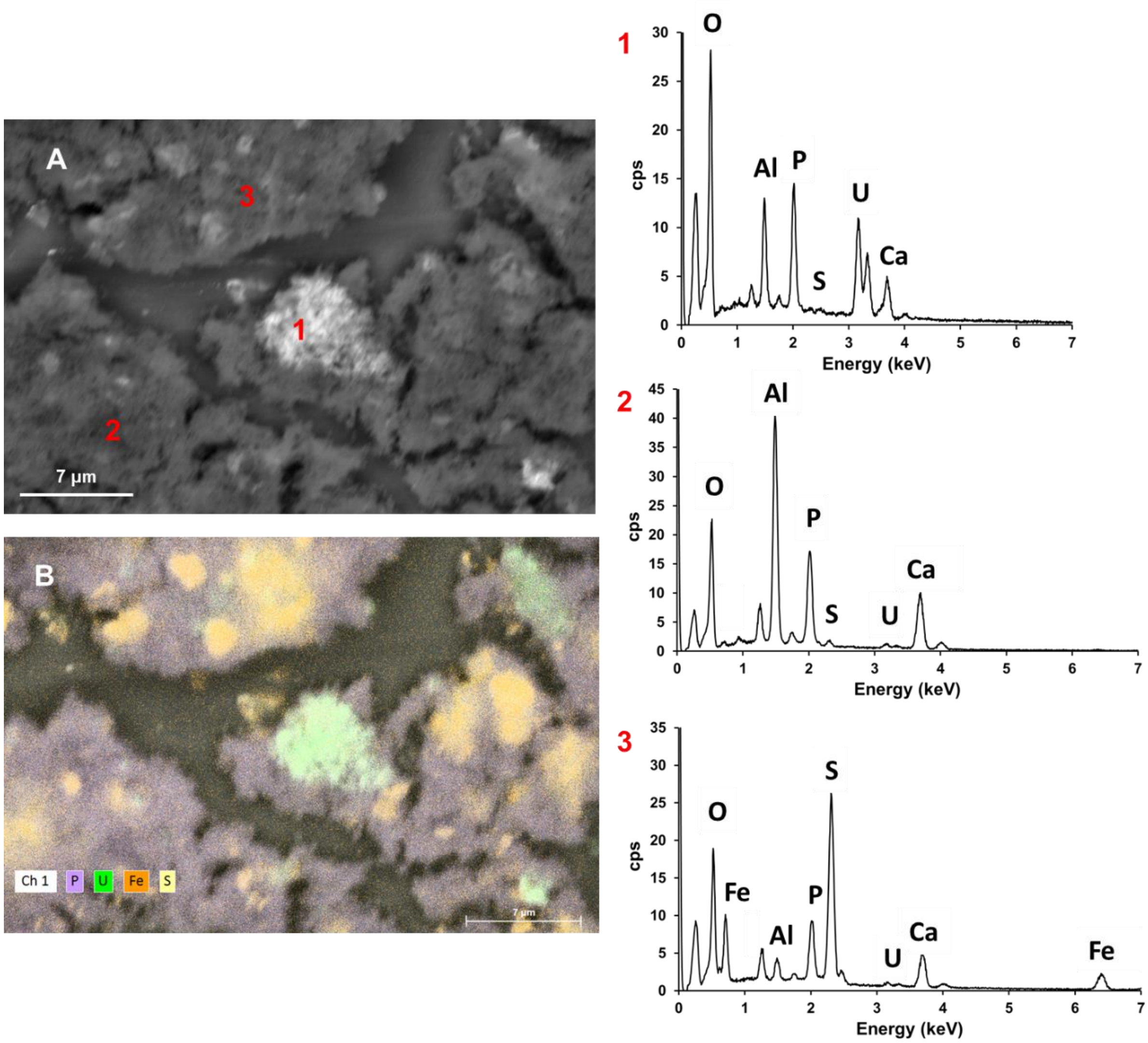

Figure S6-1 ESEM images for the low carbonate U(VI) + Fe(III) culture end point sample. (A) Backscattered image with corresponding EDS spot analysis spectra (1 - 3); (B) EDS map of the same area showing the relative distribution of P (purple), U (green), Fe (orange), and S (yellow).

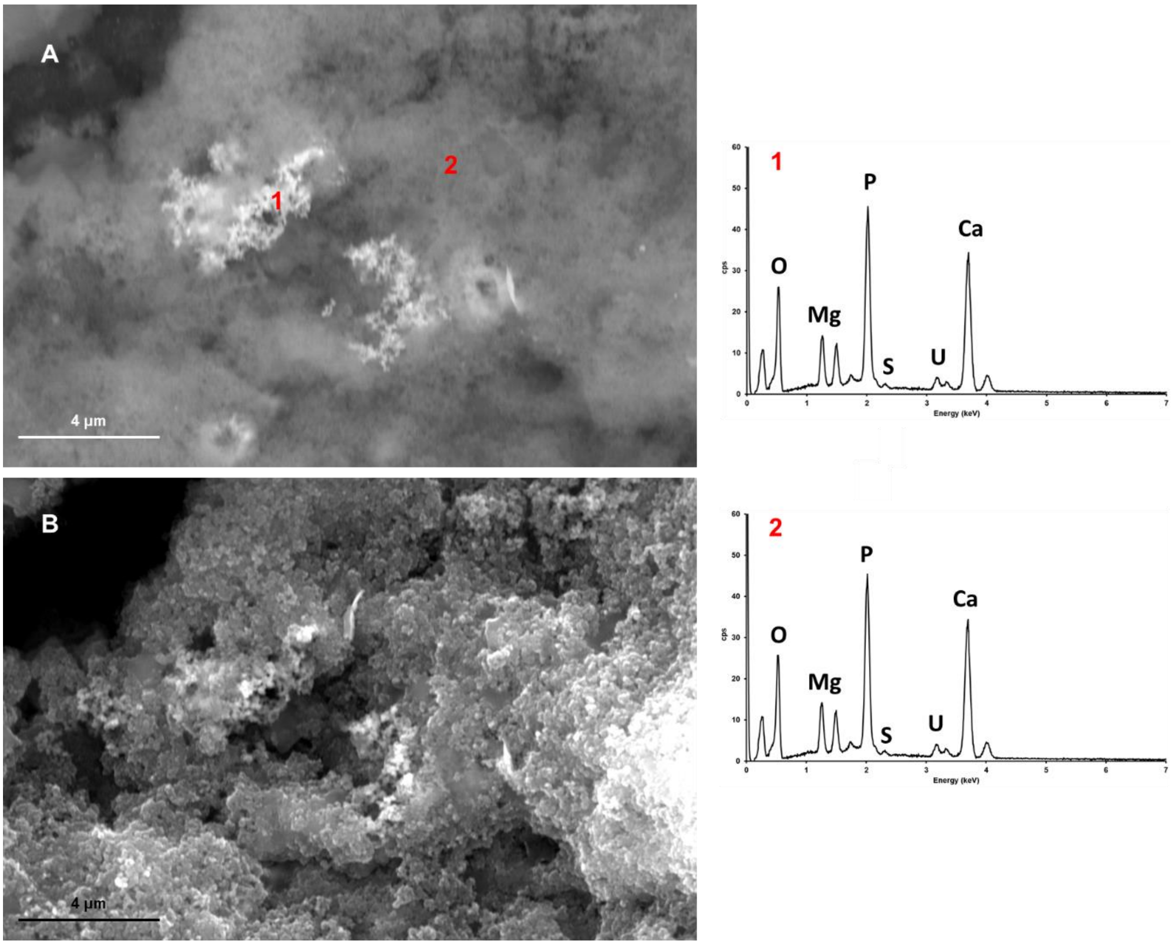

Figure S6-2 ESEM images for the low carbonate U(VI) only culture end point sample. (A) Backscattered image with corresponding EDS spot analysis spectra (1 and 2); (B) Secondary electron image showing of the same spot as (A) showing a morphology similar to that of ningyoite.

References

Alessi, D. S.; Lezama-Pacheco, J. S.; Stubbs, J. E.; Janousch, M.; Bargar, J. R.; Persson, P.; Bernier-Latmani, R., The product of microbial uranium reduction includes multiple species with U(IV)–phosphate coordination. *Geochim. Cosmochim. Acta* **2014,** *131*, 115-127.

Bargar, J. R.; Williams, K. H.; Campbell, K. M.; Long, P. E.; Stubbs, J. E.; Suvorova, E. I.; Lezama-Pacheco, J. S.; Alessi, D. S.; Stylo, M.; Webb, S. M.; Davis, J. A.; Giammar, D. E.; Blue, L. Y.; Bernier-Latmani, R., Uranium redox transition pathways in acetate-amended sediments. *Proc. Natl. Acad. Sci. U.S.A.* **2013,** *110* (12), 4506-4511.

Boyanov, M. I.; Fletcher, K. E.; Kwon, M. J.; Rui, X.; O'Loughlin, E. J.; Löffler, F. E.; Kemner, K. M., Solution and Microbial Controls on the Formation of Reduced U(IV) Species. *Environ. Sci. Technol.* **2011,** *45* (19), 8336-8344.

Beazley, M. J.; Martinez, R. J.; Sobecky, P. A.; Webb, S. M.; Taillefert, M., Nonreductive Biomineralization of Uranium(VI) Phosphate Via Microbial Phosphatase Activity in Anaerobic Conditions. *Geomicrobiol. J.* **2009,** *26* (7), 431-441.

Mehta, V. S.; Maillot, F.; Wang, Z.; Catalano, J. G.; Giammar, D. E., Effect of Reaction Pathway on the Extent and Mechanism of Uranium(VI) Immobilization with Calcium and Phosphate. *Environ. Sci. Technol.* **2016,** *50* (6), 3128-3136.

Newsome, L.; Morris, K.; Trivedi, D.; Bewsher, A.; Lloyd, J. R., Biostimulation by Glycerol Phosphate to Precipitate Recalcitrant Uranium(IV) Phosphate. *Environ. Sci. Technol.* **2015,** *49* (18), 11070-11078.

Singh, A.; Catalano, J. G.; Ulrich, K.-U.; Giammar, D. E., Molecular-Scale Structure of Uranium(VI) Immobilized with Goethite and Phosphate. *Environ. Sci. Technol.* **2012,** *46* (12), 6594-6603.
